# Motivational mediation between coping and post-traumatic growth in previously bullied college students

**DOI:** 10.3389/fpsyg.2022.1048270

**Published:** 2022-12-20

**Authors:** Yennifer Ravelo, Olga M. Alegre, Hipólito Marrero, Rosaura Gonzalez-Mendez

**Affiliations:** ^1^Instituto Universitario de Neurociencias (IUNE), Universidad de La Laguna, San Cristóbal de La Laguna, Spain; ^2^Department Psicología Cognitiva, Social y Organizacional, Universidad de La Laguna, San Cristóbal de La Laguna, Spain; ^3^Department Didáctica e Investigación Educativa,Universidad de La Laguna, San Cristóbal de La Laguna, Spain

**Keywords:** post-traumatic growth, resilience, bullying, peer victimization, BIS/BAS, regulatory focus, coping, conditional process analysis

## Abstract

Research has consistently shown that experiences of peer victimization may have long lasting negative consequences on health and academic achievement. Less attention has been paid to the association between past bullying and post-traumatic growth in college students. This cross-sectional study aims to examine the role of different motivational orientations (The Behavioral Inhibition and Behavioral Activation Systems (BIS/BAS) and regulatory focus) as potential mediators between cognitive strategies (rumination and resilient coping) and post-traumatic growth (PTG). Using a large sample of 1,134 college students, 85 were selected who were in their first year of college and had reported having previously experienced bullying. After classifying the participants acording to their the 33^rd^ and 66^th^ percentile scores on post-traumatic growth, a univariate analysis of variance (ANOVA) indicated significant differences between the low and high groups, with those highest in PTG showing the highest scores on drive approach, focus on promotion, and resilient coping. Conditional process analysis with these significant variables revealed that regulatory focus on promotion mediates between resilient coping and post-traumatic growth, whereas drive moderates the link between both variables. The findings shed light on the motivational mechanisms underlying PTG, which may be useful to guide interventions to prevent the consequences of bullying.

## Introduction

Research has consistently shown that experiences of peer victimization may have long lasting negative consequences, such as depression, suicidal thoughts, and self-harm (Lereya et al., 2015; Moore et al., 2017; Bryson et al., 2021; Van Geel et al., 2022), drug use (Ttofi et al., 2016), or externalizing problems (Reijntjes et al., 2011). In addition, academic achievement is often negatively affected in students who have been bullied (Nakamoto and Schwartz, 2009; Holt et al., 2014). However, students victimized by bullying do not always develop psychological harm, which is estimated to be approximately one in four (Montes et al., 2022). Despite this, research has paid little attention to those resilient students who function better than expected (Sapouna and Wolke, 2013; Fonseca et al., 2022), and even less to the impact of past bullying on college students’ resilience (Villora et al., 2020; Yubero et al., 2021) and post-traumatic growth (Andreou et al., 2021).

In addition to peer victimization’s association with greater vulnerability pre- and post-bullying (Woods et al., 2009; Menesini and Salmivalli, 2017), it has been suggested that some delayed outcomes of bullying may be associated with the development of distorted thoughts that make psychological and social adjustment more difficult (Mezulis et al., 2004; Zwierzynska et al., 2013). For instance, peer victimization has been related to critical self-referential attributions in the face of ambiguous peer experiences (Prinstein et al., 2005) and to non-adaptive coping strategies focused on emotions, such as self-blame (Baumgartner et al., 2021). However, the process of attempting to understand what happened and cope with peer victimization could also represent an opportunity to thrive, as in fact occurs with other forms of adversity (Cann et al., 2011).

Responses to adversity range from chronic distress to resilience, with resilient people being expected to maintain relatively stable and healthy levels of functioning, recover better, or even thrive following highly stressful experiences (Fisher et al., 2019; IJntema et al., 2021). Resilience is increasingly seen as a dynamic process that involves a positive adaptation to adverse experiences (Bonanno et al., 2015; Fisher et al., 2019; Masten, 2021), not necessarily traumatic (Feeney and Collins, 2015). Post-traumatic growth (PTG) can be considered a resilience trajectory (Fisher et al., 2019), which requires exposure to traumatic experiences and subsequent positive changes, not merely a return to previous levels of functioning (Grych et al., 2015; Vloet et al., 2017).

PTG has been defined as an enduring positive change that occurs as a result of struggling to cope with a significant life challenge (Tedeschi et al., 2004), not merely because adversity is experienced (Tedeschi et al., 2004; Calhoun and Tedeschi, 2006). Such positive changes are often reported in one or more dimensions: strengthening relationships, a greater sense of personal strengths, a greater appreciation for life, new possibilities for one’s life, and spiritual development (Tedeschi et al., 2004). However, PTG does not necessarily put an end to distress in trauma survivors (Tedeschi and Calhoun, 1995; Calhoun and Tedeschi, 1998), as it is not considered a static outcome, but an ongoing process (Tedeschi et al., 2004).

### The current study

Reviews of research on positive consequences of adversity confirm the relevance of different predictors of PTG (Henson et al., 2021). Among the cognitive strategies that significantly relate to PTG are rumination and positive coping. However, knowledge about the mechanisms underlying PTG remains limited. Hence, the interest in examining the role of different motivational orientations (approach and avoidance, and regulatory focus) as potential mediators between both cognitive strategies (rumination and positive coping) and PTG.

A large body of evidence has shown that ruminative thought contributes to intensify and extend sad moods, as well as being associated, both concurrently and prospectively, with depression and anxiety disorders (Rude et al., 2007; Aldao et al., 2010). However, while intrusive rumination about an adverse event has been found to maintain distress, deliberate rumination, aimed at understanding and problem-solving, has been proposed as a way of understanding the role of cognitive processing in PTG (Cann et al., 2011). Although there is evidence supporting that deliberate rumination contributes to post-traumatic growth (Allbaugh et al., 2016; Kramer et al., 2020; García et al., 2022), it is still necessary to examine the association between rumination and PTG in college students who have been victimized by bullying, as it has been suggested that different events could be associated with different patterns of PTG (Lowe et al., 2020).

In response to bullying, using effective coping strategies is also considered basic to build and sustain students’ resilience over time (Nixon et al., 2020). Some studies have tried to identify the most effective strategies to deal with the experience. For instance, Xie et al. (2020) found that help-seeking, avoidance, and self-defense were the most frequently used coping strategies in children. Nixon et al. (2020) identified support-seeking, use of humor, and cognitive restructuring as the most effective. In both cases, however, the effectiveness of these coping strategies depended on factors such as age, gender, forms of victimization, or repetition of peer victimization. In fact, Nixon et al. (2020) indicated that only the use of cognitive restructuring was related to lower levels of associated emotional distress.

Resilient coping is considered to be the ability to cope with stress in a highly adaptive manner (Sinclair and Wallston, 2004). It involves adopting creative ways to deal with difficulties and taking advantage of them to thrive. In addition to requiring reappraisal of negative events, this coping strategy could be most effective after highly stressful experiences such as severe peer victimization. Considering all this, both deliberate rumination and resilient coping are expected to be positively related to PTG.

*Hypothesis 1*: Students higher in PTG show higher scores in deliberate rumination and resilient coping than those lower in PTG.

Motivation underlies human behavior, cognitive processes, and emotions (Higgins, 2012). Hence, the potential of motivational mechanisms to understand resilience in the face of adversity. Two theoretical approaches have proven useful to identify distinct motivational orientations that predict resilience, albeit there are still few studies that have specifically related them to PTG (Gonzalez-Mendez et al., 2020). These approaches cover the two basic dimensions of motivation: energy and direction (Reeve, 2018). BIS/BAS represent a biopsychosocial approach to the energy displayed to overcome life challenges, which is thought to explain the construction of personality. By contrast, regulatory focus adopts a psychosocial focus to explain the direction of effort either for promotion or prevention. Interestingly, although both systems have been claimed as independent and supported by different brain networks, they are also considered complementary. Hence, evidence about their interaction in psychological processes such as resilience is necessary to support their relevance for interventions (Strauman and Wilson, 2010).

The Behavioral Inhibition System (BIS, avoidance) and Behavioral Activation System (BAS, approach) have been proposed as two major neurobiological systems that contribute to explaining people’s motivation to avoid aversive outcomes or to approach goal-oriented outcomes, respectively (Gray, 1982). Research has found that higher BAS sensitivity is positively associated with factors that predict resilience, such as sense of control (Windsor et al., 2008) or adaptive cognitive emotion regulation (Sun et al., 2020). By contrast, higher BIS sensitivity has been related to maladaptive outcomes in the face of adverse experiences (Sun et al., 2020), although this latter finding has not been consistently found (Taubitz et al., 2015; Gonzalez-Mendez et al., 2020; Masuyama et al., 2022). Given the scarcity of studies examining the relationship between BIS and PTG, it is not possible to rule out that there is a relationship between them. Therefore, BAS and BIS are expected to be associated with PTG, although in opposite directions.

*Hypothesis 2*: Students higher in PTG show higher scores in approach and lower in avoidance than those lower in PTG.

On the other hand, Regulatory Focus theory contributes to the understanding of continuous efforts in pursuit of goals (Lanaj et al., 2012). According to this theory (Higgins et al., 1997, 2001), individuals tend to focus attention on life changes and challenges as an opportunity (promotion) or as involving potential personal or social losses (prevention). These independent motivation orientations (promotion and prevention) are expected to affect the goals on which individuals focus their efforts (Higgins, 2012). Although the predictions of this approach have received extensive support in organizational contexts (Lanaj et al., 2012), the results about resilience are not always consistent. While some studies have found that both focus on promotion and prevention are related to workers’ resilience (Kuntz et al., 2017), others only highlight the association between resilience and focus on promotion (Kakkar, 2019). Among college students, evidence supports that focusing on promotion leads individuals to work to achieve good grades and the respect of peers, whereas focusing on prevention leads them to work to avoid failing grades and the disrespect of peers (Hodis et al., 2017; Gao et al., 2022). However, as far as we know, regulatory focus has not been examined to understand mechanisms underlying resilience in students that have been bullied before entering college. Despite their negative experience, those students higher in focusing on promotion are expected to show higher PTG than those higher in focusing on prevention, since they are characterized by trying to take greater advantage of the education they receive.

*Hypothesis 3*: Students higher in PTG show higher scores in focusing on promotion and lower in prevention than those lower in PTG.

Research examining the mediating (or moderating) role of approach / avoidance, or BIS / BAS, to understand how (or when) resilience occurs is still scarce. For instance, Li et al. (2019) examined the mediating role of problem-focused coping on the relationships between both regulatory focuses (promotion and prevention) and subjective well-being. However, regulatory focus seems to fit better in the role of mediating variable than coping due to its motivational character. Moreover, Toyoshima et al. (2021) found that BAS played a negative moderating role between negative life events and depressive symptoms, but they did not find a moderating effect of BIS. Based on the scant available evidence, we make similar hypotheses for approach/focus on promotion, on the one hand, and for avoidance / prevention, on the other.

*Hypothesis 4*: Resilient coping and deliberate rumination are enhanced by higher approach behaviors, which then increase PTG.

*Hypothesis 5*: Resilient coping and deliberate rumination are reduced by higher avoidant behaviors, which then decrease PTG.

*Hypothesis 6*: Resilient coping and deliberate rumination are enhanced by higher regulatory focus on promotion, which then increase PTG.

*Hypothesis 7*: Resilient coping and deliberate rumination are reduced by higher regulatory focus on prevention, which then decrease PTG.

In short, the main objective of this study is to examine the role of different motivational orientations (approach and avoidance behaviors, and regulatory focus) as potential mediators between distinct cognitive strategies (rumination and resilient coping) and PTG. As a preliminary step, we examine to what extent these motivational orientations and cognitive strategies contribute to differentiate between first-year college students with different levels of post-traumatic growth.

## Materials and methods

### Participants

Using a large sample of 1,134 college students, we selected 85 participants (77.6% women) who were in their first year of college and who had reported having experienced peer victimization before entering college. Past bullying had happened during secondary school for most (70.6%). Participants were studying different degrees at the Universidad de La Laguna, Spain. Their ages ranged from 18 to 66 (*M* = 21.5, *SD* = 6.3). From 12 to 16 years of age, which generally comprises a complete educational cycle in Spain, 70.6% claim to have suffered bullying; in the following 2 years, until entering university, 20% indicated bullying; and only 9.4% said they had experienced bullying in both periods. The average time elapsed since the last remembered bullying experience was 6.2 years (*SD =* 6.0).

### Procedure

The design of the study received the approval of the Academic Committee of the Doctoral Program in Psychology, and it was conducted in compliance with the ethical standards of the Institutional Review Board of the Universidad de La Laguna. All participants received information about the objective and procedure of the investigation and gave their consent before participating in the study. They received the links to two different questionnaires, which were completed online with a separation time of 2 months between them. Participation was totally voluntary. The anonymity and confidentiality of data were ensured at all times.

In the first questionnaire, participants were asked if they had experienced any peer victimization before entering college. If so, they were also asked to respond to a PTG scale. Once the students who had experienced peer victimization were identified and having agreed to continue participating in the study, they received a second questionnaire with the rest of the measurement items.

### Measures

An instrument was designed to collect general information on age, gender, degree, and “time elapsed” since the last episode of peer victimization that they remembered. In addition, the instrument included several scales, which are described below.

#### Post-traumatic growth

Post-traumatic growth was measured using the shortened 9-item scale from the Resilience Portfolio Measurement Packet (Hamby et al., 2015) (e.g., “I have a greater appreciation for the value of my own life,” “Now, I know that I can handle hard times”). Response options ranged from 1 (*not true*) to 4 (*mostly true*) and Cronbach’s alpha reached a value of 0.85.

#### BIS/BAS behavioral inhibition, behavioral activation

The BIS/BAS scale (Carver and White, 1994) measures both the behavioral approach system (BAS) and the behavioral inhibition system (BIS). While BAS regulates appetitive motives, whose goal is to move toward something desired, BIS is thought to regulate aversive motives, in which the goal is to move away from something unpleasant. This scale consists of 24 items. Response options range from 1 (*strong disagreement*) to 4 (*strong agreement*). Along with four filler items, the other items are grouped into seven factors. BAS Drive includes four items (e.g., “I go out of my way to get things I want,” “When I go after something I use a “no holds barred” approach”), whose internal consistency was 0.65. BAS fun seeking also consists of four items (e.g., “I’m always willing to try something new if I think it will be fun,” “I crave excitement and new sensations”) (α = 0.52). BAS reward responsiveness consists of five items (e.g., “When I’m doing well at something, I love to keep at it,” “When good things happen to me, it affects me strongly”) (α = 0.68). BIS anxiety consists of five items (e.g., “Criticism or scolding hurts me quite a bit,” “I worry about making mistakes”) (α = 0.83). BIS fear is measured with two items (e.g., “Even if something bad is about to happen to me, I rarely experience fear or nervousness,” “I have very few fears compared to my friends”) (α = 0.40). Fun-seeking and Fear were not included in the study analyses due to their low internal consistency.

#### Regulatory focus

The Regulatory Focus Questionnaire (RFQ; Higgins et al., 2001) assesses the differences in information processing using 11 items, six for measure promotion (e.g., “How often have you achieved milestones or goals that have excited you like that have excited you like to try even harder to try even harder?”) and five for prevention (e.g., “How often did you obey the rules your parents gave you?”). Response options ranged from 1 (*never or hardly ever*) to 5 (*always*). Cronbach’s α coefficients were 0.74 and 0.70 for promotion and prevention dimensions, respectively.

#### Rumination

Rumination was measured using the Event Related Rumination Inventory (Cann et al., 2011). This scale consists of 20 items that are grouped into two factors. Ten of the items measure intrusive rumination (“Thoughts about the event came to mind and I could not stop thinking about them,” “Other things kept leading me to think about my experience”), whereas the other ten refer to deliberate rumination (e.g., “I thought about whether I have learned anything as a result of my experience,” “I thought about the event and tried to understand what happened”). Participants were asked to rate the degree to which the thoughts occurred during a specified time frame from 0 (*not at all*) to 3 (*often*). Cronbach’s α coefficients were 0.92 and 0.84 for intrusive and deliberate rumination, respectively.

#### Resilient coping

Resilient coping was measured using the Brief Resilient Coping Scale (Sinclair and Wallston, 2004; BRCS), which has been adapted to the Spanish language by Tomas et al. (2021). This short scale consists of four items (e.g., “I look for creative ways to alter difficult situations,” “I believe I can grow in positive ways by dealing with difficult situations”). Response options ranged from 1 (*does not describe me at all*) to 5 (*describes me very well*). Participants were asked to rate the degree to which they felt described by each item. Cronbach’s alpha was 0.70.

### Data analysis

The Harman single factor test (Podsakoff et al., 2003) was used to confirm that a single factor accounted for less than the critical criteria of 40%. The single factor extracted from exploratory factor analysis accounted for 17.37% of the variance, indicating that the common method bias is not obvious.

To ensure comparable scale metrics for all variables, scale scores were standardized by converting them to z scores. Descriptive analyses and partial correlations of the analyzed factors were then computed while controlling for age and “time elapsed” since the last remembered peer victimization event. Participants were subsequently classified into three groups according to their 33rd and 66th percentile scores on PTG. This enabled a classification of the participants as being “low” (those who scored below the 33rd percentile, *n* = 37), “medium” (between the 33^rd^ and the 66^th^ percentile, *n* = 21), or “high” (higher than the 66^th^ percentile, *n* = 27) in PTG. Then, univariate analysis of variance (ANOVA) was conducted to examine to what extent the variables analyzed contributed to differentiate between college students with different post-traumatic growth. Previously, the homogeneity of the variances was tested using Levene’s test. Most post-hoc analyses were carried out using Bonferroni’s test. Welch’s test and the Games-Howell’s test were used in the cases in which variances were not homogeneous.

Finally, the mediating analysis was tested using the SPSS26 macro program PROCESS 4.1 (Hayes, 2022). PTG was included in the analysis as a criterion variable. The results of the ANOVA guided the choice of the mediating variables for this analysis. Gender and time elapsed since the last peer victimization experience were controlled for all the regressions. The interactions between the variables were also analyzed. Bootstrap method was used in mediation analysis to calculate 95% confidence intervals for each of 10,000 repeated samples. Statistical support for the mediation was assumed when zero was outside the confidence interval.

## Results

### Descriptive statistics and correlation analyses

Table 1 shows the descriptive statistics for all participants and partial correlations between the variables examined while controlling for age and time elapsed after the last remembered peer victimization event. PTG positively correlated with two of the BAS dimensions (drive and reward), the promotion dimension of regulatory focus and resilient coping. By contrast, PTG did not correlate with “time elapsed” since the last remembered peer victimization experience. Moreover, “time elapsed” only correlated positively with age (*r* = 0.76, *p* < 0.01) and negatively with intrusive rumination (*r* = −0.22, *p* < 0.1). Age did not correlate with any of the other variables analyzed.

**Table 1:** Partial correlations, controlling for age, and time elapsed since the last remembered bullying event, and descriptive statistics for the analyzed variables.

Variables	1	2	3	4	5	6	7	8	*M*	*SD*
1. PTG									2.70	0.70
2. Drive	0.31^**^								2.58	0.64
3. Reward	0.25^*^	0.42^***^							3.38	0.49
4. Anxiety	−0.02	−0.28^*^	0.15						3.41	0.62
5. Promotion	0.36^**^	0.49^***^	0.42^***^	−0.26^*^					3.23	0.71
6. Prevention	0.14	0.03	0.27^*^	0.01	0.33^**^				3.58	0.84
7. Resilient coping	0.30^**^	0.46^***^	0.29^**^	−0.17	0.43^***^	0.14			3.25	0.82
8. Deliberate rumination	0.06	−0.01	0.09	0.43^***^	−0.03	0.01	0.17		2.00	0.63
9. Intrusive rumination	−0.16	−0.22	−0.02	0.56^***^	−0.21	−0.08	−0.17	0.60^***^	2.13	0.72

^*^p < 0.05. ^**^p < 0.01. ^***^p < 0.001.

### Comparison between the levels of PTG

The results of ANOVAs and post-hoc comparisons are shown in Table 2. The groups classified as high and low in PTG differed significantly in resilient coping and two of the motivational dimensions, drive approach and regulatory focus on promotion. Compared with the lowest in PTG, those students who scored highest also showed higher resilient coping and no differences in deliberate rumination, thus partially confirming hypothesis 1. The highest in PTG also stood out by showing higher drive and focus on promotion, but no differences in avoidance or focus on prevention. Therefore, hypotheses 2 and 3 were only partially supported. The students with medium PTG did not differ from the other two groups in any of the variables analyzed.

**Table 2:** ANOVA and *post-hoc* analyses comparing the variables according to the levels of PTG.

	Descriptive statistic			ANOVA				
	*L*	*M*	*H*	*F*(2.82)	*η^2^*	*Post-hoc*		
	*M* (SD)	*M* (SD)	*M* (SD)			*L-M*	*L-H*	*M-H*
Behavioral activation (BAS)								
Drive	−0.35 (0.81)	0.28 (0.76)	0.26 (1.25)	5.32 ^W**^	0.10	–	−0.61^*^	–
Reward	−0.19 (1.18)	0.07 (0.83)	0.21 (0.82)	1.32^W^	0.03	–	–	–
Behavioral inhibition (BIS)								
Anxiety	0.20 (0.75)	−0.25 (1.21)	−0.10 (1.09)	1.60^W^	0.04	–	–	–
Rumination								
Intrusive	0.23 (0.87)	−0.27 (0.96)	−0.09 (1.14)	2.11^W^	0.04	–	–	–
Deliberative	−0.01 (1.02)	−0.19 (1.06)	0.16 (0.92)	0.74	0.02	–	–	–
Regulatory focus								
Promotion	−0.32 (1.02)	0.17 (0.89)	0.31 (0.94)	3.81^*^	0.08	–	−0.64^*^	–
Prevention	−0.12 (0.98)	0.18 (0.84)	0.02 (1.14)	0.64	0.01	–	**–**	–
Resilient Coping	−0.36 (0.92)	0.18 (0.72)	0.35 (1.14)	4.78^*^	0.10	–	−0.71^*^	–

L, Low Group; M, Medium Group; H, High Group. *p < 0.05. **p < 0.01. ***p < 0.001. ^W^ Welch’s *F*.

### Conditional process analysis

Based on the ANOVA results, a parallel multiple mediator model was initially estimated using Model 4 of macro program PROCESS 4.1. Resilient coping was included as the only predictor variable. Focus on promotion and drive approach were included as mediating variables. This first analysis showed an indirect effect of coping on PTG through focus on promotion (b = 0.109, *SE* = 0.057, 95%, CI = 0.023, 0.245), as well as a significant interaction between resilient coping and drive (*F*(1,78) = 6.938, *p* < 0.05). Subsequently, a conditional processes analysis (Hayes and Rockwood, 2020) was conducted to test moderated mediation (Model 15). HC4 option was used to ensure the analysis was robust against violations of homoscedasticity.

**Figure 1:**
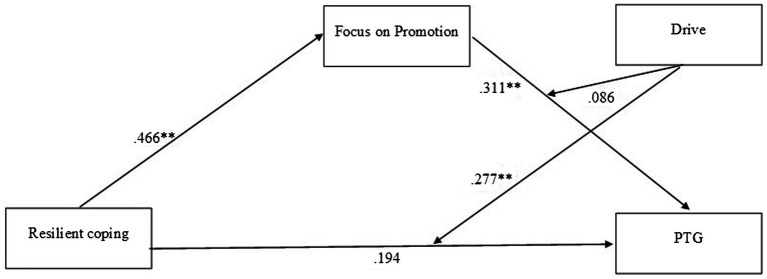
Mediating effect of focus on promotion and moderating effect of drive on the relationship between resilient coping and PTG.

As shown in Table 3, there was a positive association between resilient coping and focus on promotion (β = 0.466, *p* < 0.001), a positive association between focus on promotion and PTG (*β* = 0.331, *p* < 0.01), and a significant interaction between resilient coping and drive (*β* = 0.277, *p* < 0.01). By contrast, the index of moderated mediation was not significant, *b* = −0.042, 95% CI [−0.150, 0.054], indicating no evidence for a moderated mediation of drive. Figure 1 shows the relationships found.

**Table 3:** Regression coefficients and confidence intervals of mediation analysis.

Outcome variable	Predictor variable	Coeff	*SE (HC4)*	*t*	*p*	*LLCI*	*ULCI*	*R* ^2^	*F (HC4)*
Promotion	Coping	0.466	0.092	5.079	<0.001	0.283	0.648	0.241	9.068
PTG	Coping	0.194	0.133	1.456	0.150	−0.071	0.459	0.286	11.991
	Promotion	0.311	0.099	3.152	0.002	0.115	0.508		
	Drive	0.086	0.128	0.672	0.504	−0.169	0.341		
	Coping × drive	0.277	0.090	3.085	0.003	0.098	0.455		
	Promotion × drive	−0.091	0.102	−0.891	0.376	−0.298	0.112		
Conditional direct effects of coping on PTG at different values of drive as moderator									
	−0.915	−0.059	0.170	−0.348	0.729	−0.398	0.279		
	−0.129	0.158	0.136	1.162	0.249	−0.113	0.429		
	1.049	0.484	0.146	3.303	0.001	0.192	0.775		
Conditional indirect effects of coping on PTG, through focus on promotion, at different values of drive									
	−0.915	0.184	0.082			0.044	0.365		
	−0.129	0.150	0.060			0.051	0.287		
	1.049	0.101	0.068			−0.018	0.255		

PTG, post-traumatic growth; LLCI, lower level of the 95% confidence interval; ULCI, upper level of the 95% confidence interval; SE, Standard Error, conditional effects were calculated by PROCESS according to the 16th, 50th, and 84th percentile scores on drive.

Table 3 also shows the conditional (direct and indirect) effects of coping on PTG at different values of drive (moderating variable). The conditional direct effect of coping on PTG occurs at higher values of drive. Using the Johnson–Neyman (J–N) technique, we found that the region of significance of the conditional direct effect is located at values of drive greater than 0.239 (b = 0.260, *SE*(*HC4*) = 0.130, *t* = 1.991, *p* = 0.05, 95%*CI* = 0.000, 0.520), such that 55.29% of cases were below and 44.71% above this level. By contrast, the conditional indirect effect of coping on PTG through focus on promotion occurs at lower values of drive.

Table 4 shows the results of the Bootstrap method used to calculate 95% confidence intervals for each of 10,000 repeated samples. These results support the positive associations between resilient coping and focus on promotion (b = 0.466, 95% CI [0.271, 0.636]) and between focus on promotion and PTG (b = 0.311, 95% CI [0.126, 0.524]). The moderating role of drive between coping and PTG can also be seen (b = 0.277, 95% CI [0.067, 0.445]). Of the hypothesized indirect effects (hypotheses 4–7), the results only supported the mediating role of focus on promotion between resilient coping and PTG (hypothesis 6).

**Table 4:** Bootstrap results for regression model parameters.

Outcome variable	Predictor variable	Coefficient	BootSE	BootLLCI	BootULCI
Promotion	Coping	0.466	0.092	0.271	0.636
PTG	Coping	0.194	0.099	−0.006	0.379
	Promotion	0.311	0.101	0.126	0.524
	Drive	0.086	0.112	−0.141	0.298
	Coping × drive	0.277	0.095	0.067	0.445
	Promotion × drive	−0.091	0.103	−0.297	0.114

## Discussion

The main objective of this cross-sectional study was to examine whether different motivational orientations (approach and avoidance behaviors, and regulatory focus) play a mediating role between cognitive strategies (rumination and resilient coping) and PTG. For this purpose, we firstly analyzed to what extent these motivational orientations and cognitive strategies contribute to differentiate between college students with different post-traumatic growth levels. Although students exposed to peer victimization may equip themselves with cognitive biases that make their psychosocial adaptation more difficult (Mezulis et al., 2004), the results of this study are consistent with evidence indicating that peer victimization may also be associated with PTG in this population (Andreou et al., 2021).

According to Baumeister (2022), the self may try to change the world to fit the self or change the self to better fit the world. Although people who experience PTG often report both types of changes (Tedeschi et al., 2004), the results of this study suggest a greater willingness to try to control environmental conditions than to better fit the self at higher PTG levels. Thus, while the students highest in PTG showed propensity to make goal-directed efforts (higher scores on drive and focus on promotion), they did not stand out for their scores in prevention or behavioral inhibition orientations.

Given the high demands that college students face, it is not surprising that high motivation contributes to thriving. In fact, evidence indicates that higher levels of education are positively associated with post-traumatic growth (Henson et al., 2021). However, even those highly motivated, but still vulnerable, college students may require support in the face of the continual demands of their academic careers.

The fact that we did not find differences between the groups in avoidance also supports the idea that the highest levels of PTG stand out for the strength of their approach motivation. Motivation “is the basis for doing” (Baumeister, 2022, p. 230) and certain related protection factors, such as purpose and psychological endurance, have already been shown to contribute to resilience by maintaining the effort despite setbacks (Hamby et al., 2018).

Cognitive strategies are also thought to predict the extent to which highly stressful events impact PTG (Tedeschi et al., 2004; Henson et al., 2021). In line with the hypotheses, resilient coping is more frequent in the highest in PTG. However, the results did not support significant differences either in intrusive or deliberate rumination about past bullying.

According to most of the evidence (Allbaugh et al., 2016; Kramer et al., 2020; García et al., 2022), deliberative (but not intrusive) rumination is expected to be higher among students who score higher on PTG. Contrary to this expectation, both types of rumination failed to discriminate between the different levels of PTG. At least in part, this may be because the memory of ruminating about bullying has faded. Although these experiences may have given rise to rumination in the past, the time elapsed since the last episode of peer victimization may have contributed to reduce rumination for most. Deliberate rumination is considered to occur at a later time than intrusive rumination (Cann et al., 2011). However, the fading of memories is a common process and only discrete memories are maintained (Ganzach and Yaor, 2019). Thus, most people tend to remember the negative “peak affects” of their adverse experiences, whereas those higher in PTG also keep memories of positive “end affects” (Gonzalez-Mendez et al., 2022).

A second explanation could be due to the changes that occur in the social-contextual environment. According to Mancini (2019), improvements in psychological and social functioning may emerge from affiliative tendencies under stress, which do not require effortful internal processing or rumination. In this regard, peer victimization may have triggered changes in the school, family, and peer environment that facilitated students’ resilience. In fact, some of the changes identified in people who experience PTG are related to the strengthening of relations (Tedeschi et al., 2004). In addition to the personal resources needed to get through previous educational stages, students who have reached college may have had more support in their environment. Access to college represents itself an important change in the students’ social context that should contribute to ongoing thriving.

The use of more active coping strategies has been associated with resilience (Martinez-Corts et al., 2015; Fisher et al., 2019), and the resilient coping measure used in this study is especially sensitive to highly proactive and creative ways of dealing with difficulties. This result points to the potential of training coping strategies to promote PTG in previously bullied college students. However, the results also support that the relationship between coping and PTG is mediated through focus on promotion, i.e., focus on promotion indirectly intervenes in the effectiveness of resilient coping to increase PTG. Although the positive relationship between regulatory focus on promotion and resilience has been previously found in organizational contexts (Kakkar, 2019), this is the first study that supports its mediating role between coping and PTG in previously bullied college students. By contrast, the results do not support that the other motivational orientations (approach/avoidant behaviors and focus on prevention) mediate between coping and PTG.

Our results back the relevance of Regulatory Focus Theory in psychological research, as well as its application to the promotion of PTG in educational settings. Motivation is determined by the interplay of internal and external factors. While approach / avoidance orientations reflect dispositional energy needed to face challenges, regulatory focus is considered a more proximal motivational process (Lanaj et al., 2012). Thus, not only does focus on promotion emerge as an underlying mechanism in the PTG process, but it can also be promoted through the context responses to bullying experiences. For instance, it has been suggested that training in a flexible set of coping tools would allow students with different regulatory focus orientations to select the ones most appropriate for specific situations, thus improving their perceptions of stressful events (Delagach and Katz-Navon, 2020).

Students highest in PTG showed higher scores on both drive and focus on promotion, but drive only moderated the relationship between resilient coping and PTG. Specifically, coping directly contributes to making PTG more likely at higher values of drive, whereas focus on promotion mediates between coping and PTG as drive decreases. This result supports the idea that BIS/BAS and regulatory focus are two complementary motivational systems. Both systems have been claimed as being independent and supported by different brain networks yet complementary. According to the theory, BAS (drive) is the biopsychological system that seems to furnish energy to overcome bullying, whereas regulatory focus decisively contributes to more efficient coping to attain higher levels of PTG.

This study expands knowledge about the positive changes reported by first-year college students who have experienced peer victimization before entering college. As far as we know, this is the first study to examine the mediating role of motivational orientations between cognitive strategies and PTG. The results point to focus on promotion as a mechanism underlying the PTG process. Specifically, focus on promotion mediates between coping and PTG at lower values of drive. Moreover, the findings reveal that coping directly contribute to making PTG more likely as drive increases.

### Limitations and future directions

Although the findings partially support most of the hypotheses, it is necessary to consider some limitations of this study. First, the sample was not gender balanced, which reflects both the greater women’s representation in college as well as their greater willingness to participate in research. However, a significant group of males who have been bullied may have been left out of the study. This makes it necessary to increase the number of male participants in future research.

In a similar vein, peer victimization may have been under-reported among those college students who have experienced more serious bullying. This may have led them not to want to participate in the study. However, the sample represents 44.7% of all first-year students who reported experiencing bullying and agreed to participate in the study, which represents a significant percentage of all first-year students.

The reliability of drive (0.65) and reward (0.68) were slightly below 0.70, which recommended for scientific purposes. Therefore, it would be necessary to expand the sample in future studies to confirm the significant relationships found between these factors and PTG.

The study was cross-sectional, and it did not include indicators of psychosocial and academic functioning. This was because we did not want to overload the students so that they would agree to continue in the study. Future studies should confirm that indicators of post-traumatic growth are prospectively associated with indicators of good functioning.

### Implications for practice

This study provides evidence that regulatory focus on promotion mediates between resilient coping and post-traumatic growth in previously bullied college students. This points to proximal motivational mechanisms underlying PTG. Instead of being a distal dispositional trait, regulatory focus is considered a proximal motivational process (Lanaj et al., 2012). Moreover, regulatory focus may be enhanced through training to make PTG more likely by using cognitive strategies such as resilient coping. Hence, the need to develop specific support programs for those previously peer victimized students.

## Conclusion

This study examined the potential of different motivational orientations (approach and avoidance behaviors, and regulatory focus) as mechanisms underlying post traumatic growth in previously bullied college students. The results support that regulatory focus on promotion mediates between resilient coping and PTG, as well as the moderating role of drive between these two factors.

## Data availability statement

The original contributions presented in the study are included in the article/Supplementary material, further inquiries can be directed to the corresponding author.

## Ethics statement

The study involving human participants was reviewed and approved by Academic Committee of the Doctoral Program in Psychology & Comité de Ética de la Investigación y Bienestar Animal (CEIBA), ULL. The participants provided their written informed consent to participate in this study.

## Author contributions

All authors listed have made a substantial, direct, and intellectual contribution to the work and approved it for publication.

## Funding

This study has received funding from various sources. It has been co-financed by Agencia Canaria de Investigación, Innovación y Sociedad de la Información de la Consejería de Economía, Conocimiento y Empleo and by the European Social Fund, 2014–2020. It has also received funding from Neuro-cognitive Toolbox to assess approach-avoidance and inhibition in mental disorders (NEUCOGTOOL), and from Project PDC2021-121850-I00. Concept Test Project 2021, Ministerio de Ciencia e Innovación, Agencia Estatal de Investigación.

## Conflict of interest

The authors declare that the research was conducted in the absence of any commercial or financial relationships that could be construed as a potential conflict of interest.

## Publisher’s note

All claims expressed in this article are solely those of the authors and do not necessarily represent those of their affiliated organizations, or those of the publisher, the editors and the reviewers. Any product that may be evaluated in this article, or claim that may be made by its manufacturer, is not guaranteed or endorsed by the publisher.
